# A comparison of 16S rRNA-gene and 16S rRNA-transcript derived microbial communities in bulk and rhizosphere soils

**DOI:** 10.3389/fmicb.2025.1608399

**Published:** 2025-06-24

**Authors:** Alessandra Ceretto, Cynthia Weinig

**Affiliations:** ^1^Department of Botany, University of Wyoming, Laramie, WY, United States; ^2^Program in Ecology, University of Wyoming, Laramie, WY, United States; ^3^Department of Molecular Biology, University of Wyoming, Laramie, WY, United States

**Keywords:** protein synthesis potential, DNA ribosomal sequences, RNA ribosomal 16S, soil microbial ecology, RNA DNA ratio

## Abstract

Root exudates in a plant’s rhizosphere alters microbial community membership and activity, which can in turn alter a plant’s health and fitness. In this study we characterized bacterial community composition, using 16S rRNA-gene (DNA) sequencing to define total community membership and 16S rRNA-transcripts (RNA) to define protein synthesis potential (PSP) as a proxy of microbial activity in both rhizosphere and bulk soils of a Wyoming native plant *Boechera stricta*. Using PSP rather than total microbial membership reveals fine-scale differences in genera between the rhizosphere and control soil communities. This study found DNA community analysis alone disproportionately increased the importance of Saccharibacteria and Gemmatimonadetes phyla in the overall soil community profile, and underestimated the importance of several known root associates (Comamonadaceae, Rhizobacter, and Variovorax), which had elevated PSP in the rhizosphere soil. Thus, the use of DNA-vs. RNA-based community characterization reveals that community composition (DNA) may not completely capture community activity (RNA). Analysis of the PSP community profile also indicated elevated levels of proteins associated with carbohydrate and amino acid metabolism in the rhizosphere-associated bacteria, which may shed light on potential mechanisms by which root exudates shape the rhizosphere soil community.

## Introduction

The thin layer of soil around plant roots, called the rhizosphere, contains microorganisms that affect plant health and fitness, and these effects are sufficiently large that the rhizosphere microbiome has been described as the second genome of a plant (Berendsen et al., 2012). The rhizosphere microbiome increases plant nutrient access (Chen et al., 2002; Richardson and Simpson, 2011; Mendes et al., 2013), relieves and increases tolerance to abiotic stress (Mendes et al., 2013; Zolla et al., 2013), provides protection of a plant against disease (Mendes et al., 2011; van der Voort et al., 2016), promotes plant growth and health both directly and indirectly (Bashan, 1998; Lugtenberg and Kamilova, 2009; Zarraonaindia et al., 2015; Henning et al., 2016), and can alter the plant’s phenology, such as flowering time (Wagner et al., 2014; Panke-Buisse et al., 2015).

The majority of the rhizosphere microbial community is recruited from microbes in the surrounding bulk soil (Mendes et al., 2013; Philippot et al., 2013; Wagner et al., 2014; Zarraonaindia et al., 2015; van der Voort et al., 2016), and recruitment is driven by substrate utilization of root exudates given off by the plant, resulting in niche partitioning of soil microbes into the rhizosphere (Bulgarelli et al., 2013; Chaparro et al., 2013; Baetz and Martinoia, 2014; Huang et al., 2014; Zhalnina et al., 2018). The degree to which a plant’s rhizosphere differs from the surrounding bulk soil is called the rhizosphere effect (Philippot et al., 2013; Sasse et al., 2018), which varies by plant genotype, developmental age, life history, and numerous other factors (Lundberg et al., 2012; Bulgarelli et al., 2013; Chaparro et al., 2013; Schreiter et al., 2014; Shi et al., 2015; Pérez-Jaramillo et al., 2017).

Microbial communities may differ with regard to total microbial community membership or potential community activity. The 16S rRNA-gene (referred hereafter to as DNA) encodes for the small ribosomal subunit rRNA, which is a conserved marker gene used in many studies to rapidly characterize bacterial communities and which reflects the total microbial membership in a community (Caporaso et al., 2012; Kozich et al., 2013). The 16S rRNA-transcript (referred hereafter to as RNA) is the non-coding nucleic acid component of the small ribosomal subunit, which is essential to protein synthesis (Lindahl, 1975; Nomura et al., 1984). In addition to metabolically active cells, DNA-derived community characterization can also reflect the presence of DNA from dead or lysed cells, extracellular free DNA, and dormant cells that may not be significantly active within a community (Hamilton et al., 1968; Lorenz and Wackernagel, 1987; England et al., 2004; Bakken and Frostegård, 2006). RNA molecules degrade more quickly than DNA (Karl and Bailiff, 1989), therefore RNA-based community characterization will exclude these inactive players from analysis. Community analysis using DNA and RNA allow for the calculation of RNA: DNA ratios (referred to hereafter as 16S-ratio), which normalize the concentration of RNA ribosomes by the abundance of DNA gene copies and have been used by many studies to estimate recent microbial activity (e.g., Muttray and Mohn, 1999; Zhang et al., 2014; Denef et al., 2016; Bowsher et al., 2019). RNA indicates a population’s potential to catalyze protein synthesis, via the presence of ribosomes, not the realized function and outcome of protein synthesis, therefore a more accurate descriptor of RNA and 16S-ratios is protein synthesis potential (PSP), or potential activity, rather than recent microbial activity (Blazewicz et al., 2013).

RNA-derived microbial community diversity is shown to respond more sensitively to shifting local abiotic and biotic conditions than DNA-derived community characterization (Hunt et al., 2013; Charvet et al., 2014). Among the abiotic factors changing in the rhizosphere, root exudates have been shown to alter microbial community activity and functions in addition to community membership (Hanson et al., 2008; Stuart Chapin et al., 2009; Eilers et al., 2010; Shi et al., 2011). Several studies have shown a significant increase in microbial activity in the rhizosphere due to root exudates (Drake et al., 2011; Goldfarb et al., 2011; Phillips et al., 2011; Shi et al., 2011), and it is theorized that as much as 30–50% of activity in the soil is fueled by recent root exudates generated by photosynthesis (Högberg et al., 2010; Bradford et al., 2012). Using RNA to visualize protein synthesis potential for community analysis may reveal more nuanced differentiation between soil environments in comparison to DNA-derived community characterization.

Though many studies have investigated the differences between bulk and rhizosphere soils using DNA-derived community characterization (e.g., Sharma et al., 2005; Aira et al., 2010; Mendes et al., 2011; Lundberg et al., 2012; Philippot et al., 2013; Guyonnet et al., 2018; Ma et al., 2019; Tkacz et al., 2020; Liu et al., 2022), only a few have used both DNA- and RNA-derived communities in crop species (grains and legumes in Sharma et al., 2005; rice paddy soils in Liu et al., 2019). The relative importance of differences in total microbial membership vs. protein synthesis potential in bulk and rhizosphere soils is even less well understood for wild plant species (Vieira et al., 2020). Using the short-lived perennial *Boechera stricta* grown in native sites, this study characterizes the total microbial membership and protein synthesis potential of bacterial communities in rhizosphere and bulk soils, by comparing 16S-transcript (RNA) and 16S-gene (DNA) generated amplicons. We hypothesized that the community profiles of rhizosphere and bulk soils would be more distinct from one another when looking at protein synthesis potential (RNA-derived) than total membership (DNA-derived) communities. To address this hypothesis, experimental *B. stricta* were planted in the field along with control pots containing soil of similar composition with no plants. Soil was harvested from both treatments as well as bulk soils directly from the plots over the course of 3 days, and the soil bacterial community composition was investigated at both RNA and DNA levels using Illumina high-throughput sequencing. We also investigated two methods of estimating PSP, by using 16S-ratios and wholesale analysis of the RNA-derived community. Hypothetical functional profiles of taxa differing between soil environments (rhizosphere vs. control soils) were generated to elucidate potential metabolic pathways that might be more prevalent in one environment vs. the other.

## Methods and materials

### Plant material and growth conditions

We tested for differences in community characterization based on 16S RNA *vs*. DNA biomarker sequence patterns in the rhizosphere of *B. stricta*, a perennial herb native to Wyoming. Seeds for this study were originally collected from the Snowy Range Mountains (41.32971759902109 N, -106.50515422710646 W), and grown for one generation in the greenhouse to increase seed numbers and minimize maternal effects. Prior to planting, seeds were surface sterilized, by rinsing for 1 min in 70% ethanol 0.1% Triton 30% RO water mixture, then rinsed in RO water, then rinsing for 12 min in a 10% bleach 0.1% Triton 90% water mixture. Seeds were then rinsed a final three times with RO water, before being placed on sterile filter paper for ease of planting (adapted from Lundberg et al., 2012).

Surface-sterilized seeds were planted into pots with a mixture of field and potting soils. For the field soil, we collected soil from unvegetated sites adjacent to a field location with a native *B. stricta* population, referred to hereafter as the Crow Creek field site (CRW). Field soil was sieved to 4 mm to remove large debris, then autoclaved three times for 30 min with the soil being mixed between autoclaving steps. This soil was then mixed with autoclaved potting soil [Redi-Earth Potting Mix (Sungro Horticulture, Agawam, MA, United States)] in a 9:1 ratio; we included a small percentage of potting mix because its greater water-holding capacity relative to the field soil improves the overall rate and synchrony of seed germination. This soil mixture was next inoculated with a 4% v/v of non-autoclaved field soil inocula. This approach of autoclaving all soil and then applying a field soil inoculation was used to ensure that detected microbes were those native to *B. stricta* and not derived from the potting mix.

Before field transplanting, seeds germinated in 2″ mesh net pots (2″ Inch TEKU Net Slit Pots for Hydroponic Aeroponic Use) and were allowed to grow for 4 weeks under greenhouse conditions (UW Laramie Research and Extension Center, Laramie, WY) with ambient day/night light and temperature cycles. In addition to pots planted with seeds, which would be used to characterize the rhizosphere microbiome, we also prepared soil-filled pots without plants, which would be used to estimate the microbiome of bulk soil or unvegetated microsites, referred to hereafter as the “control soil.” Pots were placed in a randomized checkerboard array into tray blocks, such that no pot was directly adjacent to another. Plastic covers were placed over all trays to retain humidity and promote germination. All materials for planting, such as bench tops, trays, pots, and covers were bleached and rinsed before use. Pots were individually watered, initially via subirrigation and after 2 weeks of growth via overhead misting. Covers were removed 2 weeks after germination. Three weeks after germination, all pots were acclimated to the outside environment via 2-h field exposures. Plant germination time, rosette size, and true leaf number were measured weekly to estimate plant performance.

In June of 2019, 4 weeks after germination, plants were transferred to the Crow Creek field site, which has a naturally occurring population of *B. stricta*. The Crow Creek (CRW) field site was located in the Medicine Bow-Routt National Forest in south-eastern Wyoming (41.227318 N, -105.383343 W), with an elevation of ~2,560 m. Six 26 cm by 140 cm plots were cleared of plants and debris. Mesh pots were randomized into each plot and planted roughly 10 cm apart in two rows of 24 pots. Mesh pots were removed from filters and cups and placed directly in field site plots, to minimize root damage while transplanting and to facilitate collection of plant rhizospheres. Rosette size and true leaf number were measured weekly to estimate plant performance. All pots were watered every other day using RO water, and checked for insect damage.

### Sample collection and processing

Four weeks after being transplanted to the field site, soil and plant samples were harvested. Rhizosphere soil, bulk soil, and control soil samples were collected over the course of 3 days, from July 15 2019 to July 17 2019 between 1:30 p.m. and 2:30 p.m. At each collection time point, pots with and without plants were randomly harvested and bulk soil was collected directly from within each plot. Samples from the three treatments types were handled as follows: The large samples of soil collected in the field from (1) the control pots without plants and (2) the bulk soil were stored in whirlpacks (Whirl-Pak^®^ Bags); smaller subsamples of these soils were taken and stored in 2 mL test tube for later nucleic acid extraction. (3) For collection of rhizosphere soil from the pots with plants, mesh pots were removed from the soil, with care taken to ensure that roots which had grown out of the mesh pots were damaged minimally. Plants were then removed from the pots and shaken to remove excess loose soil. Soil that adhered closely at approximately 1 mm to the root mass was considered to be rhizosphere soil. Roots and adhered soil were separated from plant leaves and stem using flame sterilized scissors, and placed in falcon tubes containing approximately 200 mL of PBS buffer (200 ul silwet, 900 mL RO water, 100 mL 10 × PBS) (adapted from Bulgarelli et al., 2013). Samples were stored on ice in the field. Immediately upon returning to the lab, bulk and control soil samples were transferred to a-80°F freezer. For subsequent sample processing, falcon tubes containing rhizosphere soil and plant root tissue were defrosted, then lightly vortexed to remove adhered soil from root tissue. Root tissue was removed using sterile forceps. The soil slurry was then vacuum filtrated through a 0.2 nm filter. The resulting soil and filter were transferred to a 0.5 mL centrifuge tube, and flash frozen with liquid nitrogen, before being stored long term at −80°C.

### Nucleic acid extraction and amplicon library preparation

During the extraction process, we took several steps to minimize biases that might artificially inflate the differences between RNA- and DNA-derived community composition measurements. Methodological biases were minimized through the use of technical replicates, and the simultaneous extraction of nucleic acids from a single soil sample, so each paired DNA and RNA community profile derives from an identical collection (Moeseneder et al., 2005; Gentile et al., 2006; Morgan et al., 2010; McCarthy et al., 2015). Some methodological differences were unavoidable, such as RNA but not DNA having a reverse transcription step (Zhen et al., 2015). Control, bulk, and rhizosphere soil RNA and DNA samples were simultaneously extracted using the methods described in the RNeasy PowerSoil Total RNA Kit and RNeasy PowerSoil DNA Elution Kit (Qiagen, 2017). After extraction, RNA was reverse transcribed to cDNA using the QuantiTect Reverse Transcription Kit (Qiagen, 2009). Negative control blank samples were included for extractions and reverse transcription. Samples were stored at −20°C until further processing.

Positive control (a ZymoBiomics mock community) and negative control (blank-H_2_O) samples were included in library preparation. 16S rRNA-gene DNA and 16S rRNA-transcript cDNA amplicons were amplified using the 515–806 (Walters et al., 2016) primer pair to amplify the V4 region of the 16S rRNA locus. Two technical replicates were made for each sample; non-blank samples were normalized to a standard concentration of 10 ng/ul. Kapa HiFi Hot Start polymerase, Kapa HiFi Hot Start buffer and reagents, and HPLC grade water were used during PCR. PCR conditions for the first round were: 95° for 3 min; followed by 15 cycles of 98° for 30 s, 62° for 30 s, and 72° for 30 s; with a final 72° elongation step for 5 min and a 4° hold. PCR products were cleaned using AxyPrep MagBead magnetic beads (Axygen; Union City, CA, United States). PCR conditions for the second round were: 95° for 3 min; followed by 19 cycles of 98° for 30 s, 55° for 30 s, and 72° for 30 s; with a final 72° elongation step for 5 min and a 4° hold. Products from the second round of PCR were also cleaned using AxyPrep MagBead magnetic beads. Library amplification success was confirmed using a Bioanalyzer fragment analyzer (Agilent; Santa Clara, CA, United States). PCR amplicon libraries were sequenced by Psomagen (Rockville, Maryland, United States) on an Illumina NovaSeq 6000 using 2 × 250 paired-end sequencing.

### Bioinformatic analysis

A custom perl script (created by C. Alex Buerkle) was used to demultiplex sequence data, and unique reads were dereplicated using vsearch v.2.9.0 (Edgar, 2010). Identified reads were clustered using the “cluster_unoise” (Quast et al., 2015) algorithm and a 99% similarity threshold, and sequences that occurred 12 or more times were considered a potential OTU. Chimeric sequences were removed using “uchime3_denovo” algorithm (Edgar, 2010), and the resulting OTU were used to make an OTU table using the “usearch_global” algorithm. Taxonomy was assigned to each OTU using vsearch v 2.15 and the Silvia v123 (Quast et al., 2015) reference database, with a minimum bootstrap confidence of 80%. Computing was performed using the Teton Computing Environment at the Advanced Research Computing Center, University of Wyoming, Laramie.^1^

Reads in negative control samples that occurred in low frequency (i.e., less than 100 reads per sample) in other samples were considered contamination and removed from the data set. Reads that did not assign to the kingdom bacteria were removed. Samples with less than 30,000 total reads were removed from analysis, and samples were filtered so each RNA derived sample had a corresponding DNA sample that was extracted from the same collected soil. OTU that occurred in less than 10% of all samples with less than 2 reads were removed. To minimize uninformative noise, OTU with less than 100 total reads across all samples were grouped together into a single taxonomic group. A total of 29,667 OTU consisting of 908,171 reads (1.2% of total reads; an average of 30 reads per OTU) were merged into a single OTU and labeled “Low Abundance OTU Group.”

The final data set consisted of 90 total samples; 36 rhizosphere soil, of which 18 were DNA derived and 18 RNA derived, 26 control soil samples, of which 13 were DNA derived and 13 RNA derived, and 28 bulk soil samples, 14 of which were DNA derived and 14 RNA derived. These samples contained 17,896 unique OTU, and 33,732,811 total reads (64% RNA reads: 36% DNA reads). Notably, selecting an even number of samples at random from each treatment (rhizosphere, control, or bulk) did not alter the conclusions of the analysis, and we therefore present results based on all 90 samples.

### Data analysis

Samples were rarified to 32,997 reads per sample, and alpha diversity was estimated using the phyloseq package “estimate_richness.” Statistical differences between community nucleic acid derivation and soil type were determined using ANOVA (analysis of variance). To calculate beta diversity, we used the package Phyloseq v1.30.0 (McMurdie and Holmes, 2013). Reads assigned to an OTU in a sample were divided by the total number of reads per sample to calculate within site proportional abundances. MDS plots were used to visualize Bray-Curtis pairwise dissimilarities of community data. Significant differences between DNA and RNA derived communities, as well as the three soil types within and between communities were determined using PERMANOVA (pairwise adonis testing with Bonferroni correction). The corncob package (Martin et al., 2020) was used to estimate differential abundances between the DNA and RNA-derived communities, and between the DNA-derived and RNA-derived rhizosphere, bulk, and control soil samples, using the absolute abundance of reads, rather than the proportional abundances. Differentially abundant taxa with *p*-values < 0.01 were considered statistically significant.

*T*-tests of 16S-ratios and differential abundance analysis of the RNA-derived communities are two methods which can be used to determine elevated levels of RNA-derived reads, and therefore protein synthesis potential (PSP), between groups. In this study, we compared both analyses for overarching patterns of PSP in the microbial communities (see Supplementary Tables S1, S2). 16S-ratios were generated by normalizing the number of RNA reads (representing the number of ribosomes in a cell) by the number of DNA reads (representing the number of gene copies that code for ribosomes in a cell), using method 3 outlined in Bowsher et al. (2019). Though 16S-ratios are a common method of estimating overarching patterns of potential microbial activity in a community (Steven et al., 2017), we focused primarily on analyzing the RNA generated data, due to biases associated with 16S ratios such as (1) the within cell variability 16S rRNA-gene copy number in different taxa, causing over estimations of the 16S-ratio, which cannot be corrected for (Schaechter et al., 1958; Cooper and Helmstetter, 1968; Klappenbach, 2001; Lee et al., 2009; Franklin et al., 2013; Louca et al., 2018), and (2) using a single arbitrary cutoff point of 16S-ratio activity, which is problematic in diverse microbial communities and may include dormant microbes in analysis (Jones and Lennon, 2010; Blagodatskaya and Kuzyakov, 2013; Blazewicz et al., 2013; Steven et al., 2017). Mean 16S-ratios were calculated for each genus (Bowsher et al., 2019), in order to be compared to the genus-level differential abundance analysis output from corncob. We again used corncob to estimate RNA-based differential abundance. Though there are some concerns associated with directly using RNA as an indicator of PSP (outlined in depth in Blazewicz et al., 2013) corncob corrects for errors inherent in microbial data analyses. Corncob correlates taxa to covariates of interest and infers possible taxa presence in samples with small sequencing depth, with calculated variance around that inference, thereby reducing sampling bias (Willis, 2019; Martin et al., 2020). It is likely that this analysis better reflects which genera have a higher number of RNA reads compared to DNA, and therefore which genera have higher PSP in the different soil types. This study therefore focuses on reporting the RNA-derived community results, with the 16S-ratio analysis as Supplementary material.

**Figure 1:**
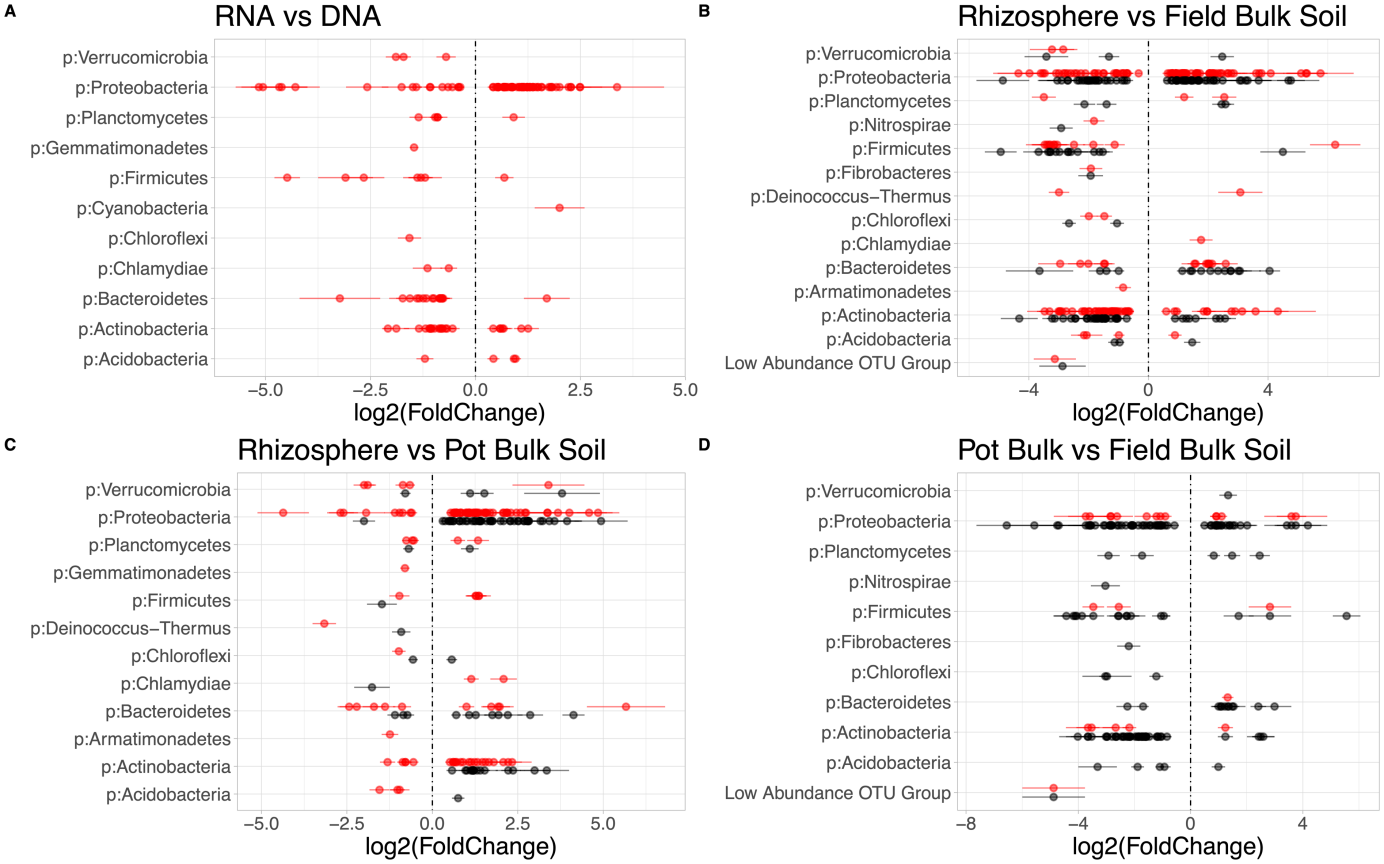
Corncob differential abundances calculated at the genus level. Each line represents a phylum, and each point a unique genus within that phylum, with each line through a point representing the standard error around each point. Each genera has differential abundance significantly different *p*-value < 0.01 between indicated community. Red colored points above the line represent genera derived from the RNA community, and black colored points below the line represent genera derived from the DNA community. **(A)** Compares abundances of RNA-derived (red) genera to DNA-derived genera, with zero being the baseline DNA. Genera above zero were more abundant in RNA community than DNA community, and genera below zero were less abundant in the RNA community. **(B)** Compares genera in the rhizosphere and bulk soils, within the RNA-derived (red) and DNA-derived (black) community, with zero being the baseline bulk soil genera the rhizosphere genera are compared to. **(C)** Compares genera in the rhizosphere and control soils, with zero being the baseline control soil genera the rhizosphere genera are compared to. **(D)** Compares genera in the bulk and control soils, with zero being the baseline bulk soil genera the rhizosphere genera are compared to.

Hypothetical functional gene profiles were created using the package Tax4Fun (Aßhauer et al., 2015), the output of which shows what percentage of the sample being analyzed is associated with a known gene and function. The RNA-derived genera that were significantly different in abundance among the soil environments in the corncob analysis were included in the Tax4Fun analysis, as were genera with a mean 16S-ratio value over one deemed significantly different between soil types using t-tests. For the Tax4Fun analysis, taxonomy of the OTU were reassigned using the Silva123 library (Quast et al., 2015) to ensure data compatibility. Gene profiles were compared to the KEGG Orthology database (Kanehisa et al., 2016) to determine the metabolic pathways with which the identified genes were associated. Mean Tax4Fun abundances were calculated for each genus in the different sample types, and the differences between genera identified as enriched *vs*. depleted were calculated to determine whether a hypothetical gene was more abundant in a certain soil treatment compared to the other. For example, in taxa identified as significantly different between rhizosphere *vs*. control soils, relative gene abundances were calculated for genera depleted in the rhizosphere and genera enriched in the rhizosphere soil. Genes with a positive value were more abundant in the “enriched (corncob −log2(FoldChange) > 0, or 16S-ratio *p* < 0.01 & *t* > 0)” rhizosphere soil category, and genes with a negative value were more abundant in the “depleted (corncob −log2(FoldChange) < 0, or 16S-ratio *p* < 0.01 & *t* < 0)” rhizosphere soil category (Figure 1).

## Results

### Sequencing results

After quality filtering and removal of chimeras, an average of 374,809 reads per sample were retained with 17,896 unique taxa in 90 samples (45 DNA, 45 RNA). Of those taxa 17,851 were present in the RNA community (16 taxa unique to RNA), and 17,880 in the DNA community (45 taxa unique to DNA).

### RNA vs. DNA microbial diversity

**Figure 2:**
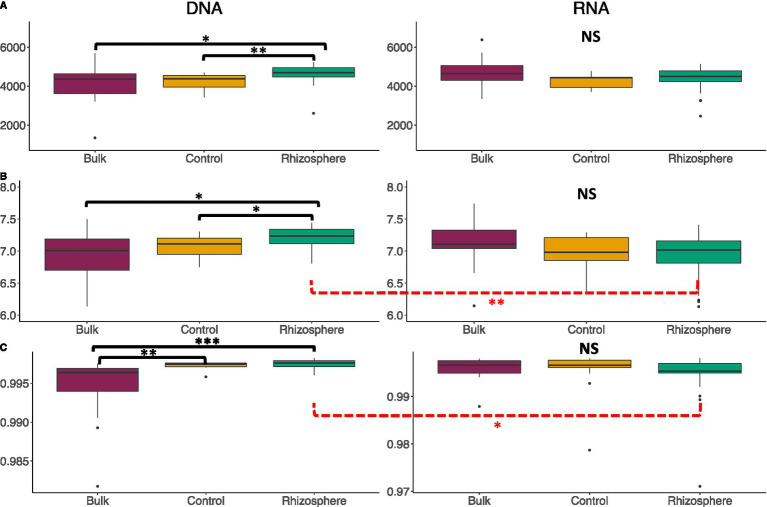
Alpha-diversity metrics for DNA- and RNA-derived community soil type comparison. Columns indicate the community being investigated (DNA-derived soil types, RNA-derived soil types), and rows indicate the analysis being used (**A**: Observed Richness, **B**: Shannons Diversity Indices, **C**: Simpsons Diversity Indices). Lines in the boxes indicate the median, with the top and bottom of boxes representing 75th and 25th quartiles. Whiskers represent the 1.5× inter quartile range (IQR). Stars indicate significant differences between community and soil types, with the black lines above the graph indicating significance between soil types within the RNA or DNA groups, and the dashed red lines below the graphs indicating significance of a soil type between the RNA and DNA groups (NS = *p*-value > 0.05, * = *p*-value < 0.05, ** = *p*-value < 0.01, *** = *p*-value < 0.001, **** = *p*-value < 0.0001).

Using rarefied communities, alpha diversity did not differ significantly between the communities characterized by DNA and RNA, in richness, Shannon, or Simpsons diversity on average between the bulk soil or control treatments. However, between the DNA and RNA, rhizosphere soils differed significantly from one another in Shannon (*p* < 0.01**) and Simpsons diversity (*p* < 0.1*) (Figure 2), indicating that DNA vs. RNA characterization capture different diversity among member *vs*. potentially active microbes.

**Figure 3:**
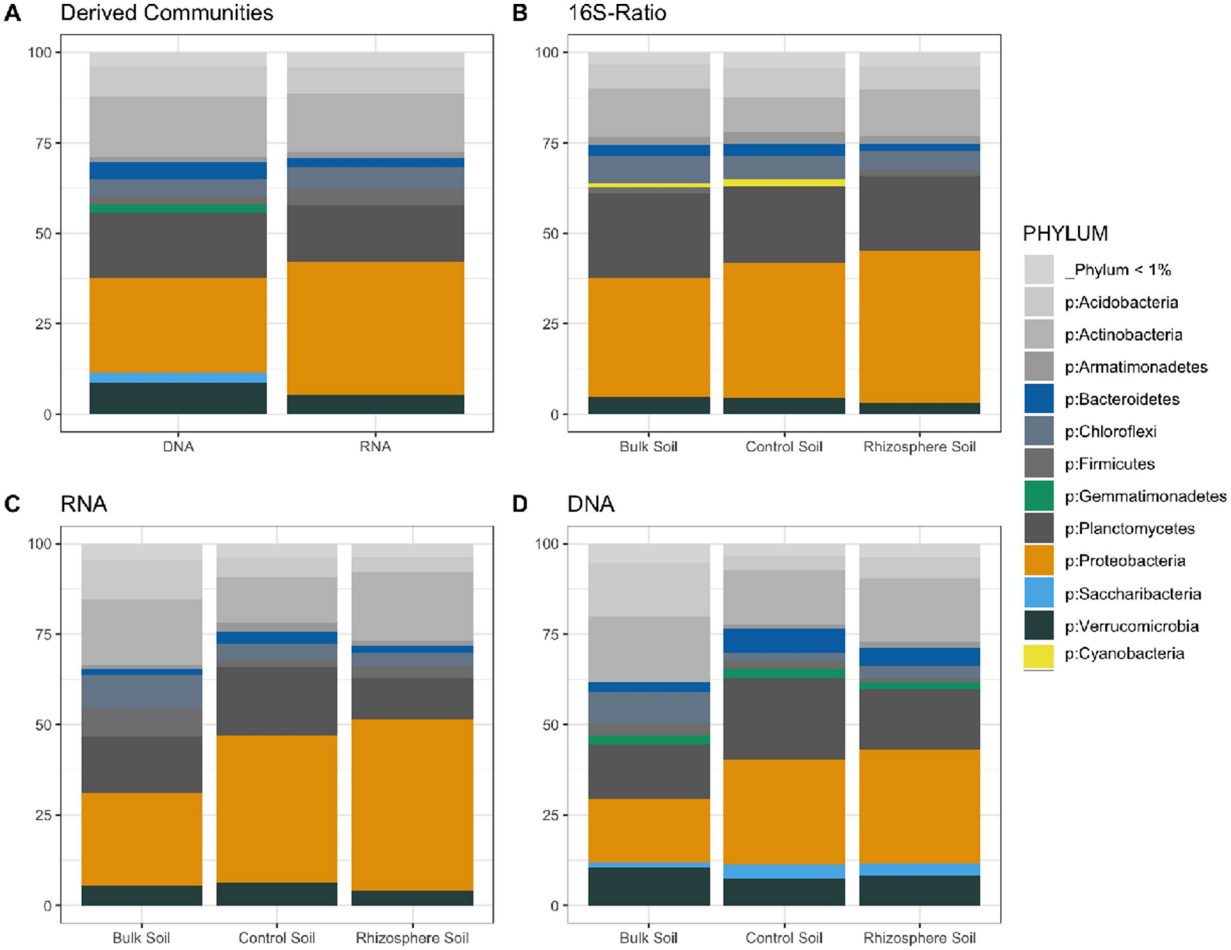
Relative abundance of phyla within a community type. Phyla with notable differences between soil types and derived nucleic acids are colored, and 15 phyla with <1% total reads in the community were pooled together into a single group. **(A)** Comparison of phyla between the RNA-derived and DNA-derived communities. **(B)** Relative activity of each phyla within bulk, control, and rhizosphere soil types, as determined by OTU 16S-ratio. **(C)** Relative abundance of phyla in the RNA-derived community. **(D)** Relative abundance of phyla in the DNA-derived community.

**Figure 4:**
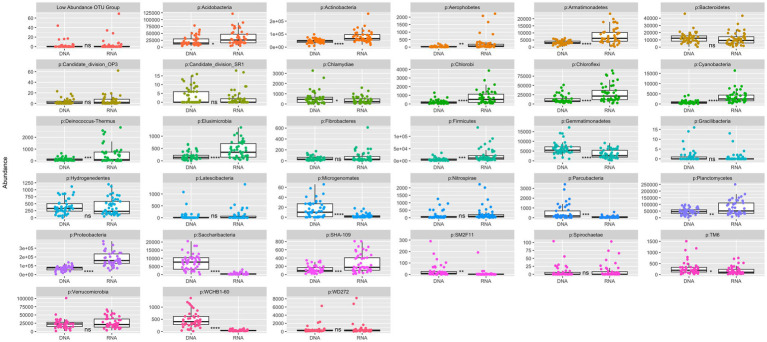
Absolute abundance of phyla compared between entire RNA and DNA communities. Lines in the boxes indicate the median, with the top and bottom of boxes representing 75th and 25th quartiles. Whiskers represent the 1.5× inter quartile range (IQR). Stars indicate significant differences between community and soil types, with the black lines above the graph indicating significance between soil types within the RNA or DNA groups, and the dashed red lines below the graphs indicating significance of a soil type between the RNA and DNA groups (NS = *p*-value > 0.05, * = *p*-value < 0.05, ** = *p*-value < 0.01, *** = *p*-value < 0.001, **** = *p*-value < 0.0001).

Visualization of the relative abundance of some representative phyla between the DNA and RNA derived communities showed differering patterns of PSP vs. microbial membership. Bacteriodetes was not significantly differ between communities, indicating an equal amount of PSP for the number of cells present in the community (Figures 3A, 4). Two phyla (p: Gemmatimonadetes, p: Saccharibacteria) were prevalent in the DNA-derived community compared to the RNA-derived community (Figures 3A,C,D), indicating little to no PSP for how present the two phyla were in the community. Proteobacteria showed significantly greater number of RNA- to DNA-derived reads, indicating a high level of PSP for number of cells present in the community (Figures 3A, 4). Visualization of the absolute abundance of phyla between the DNA and RNA derived communities show 21 phyla as being significantly different in abundance between the RNA and DNA communities, with 13 of those significantly more abundant in the RNA-derived community and the remaining 6 more abundant in the DNA-derived community (Figure 4). Visualization of the 16S-ratio PSP compared to the RNA-derived community (which indicates PSP) showed similar patterns of phyla abundance, though 16S-ratios showed a much higher PSP of p: Cyanobacteria (Figure 3B), despite making up less than 1% of both the total RNA- and DNA-derived community (Figures 3B,C).

**Figure 5:**
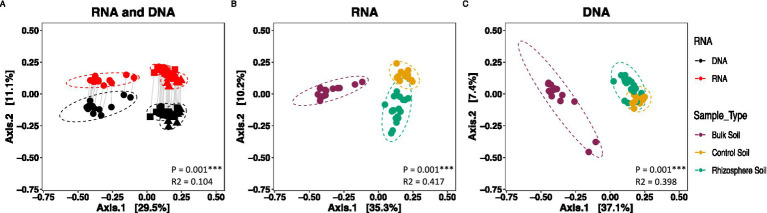
Metric multidimensional scaling (PCoA) plot of bacterial community based on Bray-Curtis dissimilarities. **(A)** RNA-derived vs. DNA-derived community. Solid gray lines connect each RNA derived sample with its corresponding DNA sample. Shape represents soil type of origin (circle = Bulk Soil, square = Control Soil, triangle = Rhizosphere Soil), and color denotes nucleic acid of origin (red = RNA, black = DNA), **(B)** RNA-derived bacterial community, **(C)** DNA-derived bacterial community. All points were normalized by abundance within a sample. Points represent unique samples, with the color representing soil sample type (purple = Bulk Soil, yellow = Control Soil, green = Rhizosphere Soil). Ellipses represent the 95% confidence interval of the mean for each soil type.

Based on Bray-Curtis analysis of the normalized data, beta diversity was significantly different between the overall DNA- and RNA-characterized communities (*p* < 0.001***; R2 = 0.104) (Figure 5A). Differential abundance analysis via corncob identified 11 phyla (137 genera) that differed in abundance based on whether they were characterized via DNA or RNA (*p* < 0.01). Of these, 71 genera were more abundant in the RNA- compared to the DNA-derived community estimation (Figure 1A).

### Soil environment comparison

The three soil environments compared in this study were rhizosphere soil, control-bulk soil with a similar physical matrix as the soils that plants were grown in, and bulk soil taken directly from the study site.

Some expected artifacts of RNA- and DNA-derived community analysis were found, though these made up <1% of the total reads used for analysis and the three soil types do not significantly differ in OTU unique to each soil type. In the DNA-derived community reconstruction 62 taxa and 3,286 reads (<1% of DNA reads) were unique to the rhizosphere soils, 12 taxa and 64 reads (<1% total DNA reads) were unique to control soils, and 256 taxa and 12,016 reads (<1% of total DNA reads) were unique to the bulk soils. In the RNA-derived community 38 taxa and 227 reads (0.1% of RNA reads) were unique to the rhizosphere soils, 76 taxa and 629 reads (<1% total RNA reads) were unique to control soils, 245 and 15,765 taxa and reads (<1%% of total RNA reads) were unique to the bulk soils.

Within the DNA-derived community, significant differences were seen between the bulk and rhizosphere soils (*p* < 0.1*) and control and rhizosphere soils (*p* < 0.01**) in richness, between the bulk and rhizosphere soils (*p* < 0.1*) and control and rhizosphere soils (*p* < 0.1*) in Shannon Diversity Indices, and between the bulk and rhizosphere soils (*p* < 0.0001***) and bulk and control soils (*p* < 0.01**) in Simpsons Diversity Indices (Figure 2). Based on Bray-Curtis analysis of normalized data from the DNA-derived community, the three soil environments were significantly different from each other (*p* < 0.001***, R2 = 0.398) (Figure 5D). Differential abundance analysis via corncob identified 144 genera that differed significantly between rhizosphere and bulk soils, with 128 genera being more abundant in rhizosphere than bulk soils (*p* < 0.001***) (Figure 1B). Between rhizosphere and control soils, 83 total genera were differentially abundant, with 73 being more abundant in the rhizosphere than control soils (Figure 1C). Between the control and bulk soils, 155 total genera were differentially abundant, with 135 genera more abundant in the control than bulk soils (Figure 1D).

Within the RNA-derived community composition, no significant differences were seen between the three soil communities in terms of richness, Shannon Diversity, or Simpson Diversity (*p* = 0.185) (Figure 2). Based on Bray-Curtis analysis of normalized data from the RNA group, the three soil environments were significantly different from each other (*p* < 0.001***, R2 = 0.4169) (Figure 5C). Differential abundance analysis via corncob identified 168 genera that differed significantly between rhizosphere and bulk soils, with 120 genera being more abundant in rhizosphere than bulk soils (*p* < 0.01) (Figure 1B). Between rhizosphere and control soils, 113 total genera were differentially abundant, with 79 more abundant in the rhizosphere than control soils (Figure 1C). Between the control and bulk soils, 179 total genera were differentially abundant, with 129 genera being more abundant in the control than bulk soils (Figure 1D). *T*-tests of significant differences between 16S-ratios of genera between soil types conflicted with corncob analysis of RNA-derived communities (Supplementary Table S1).

### Hypothetical gene profiles

**Figure 6:**
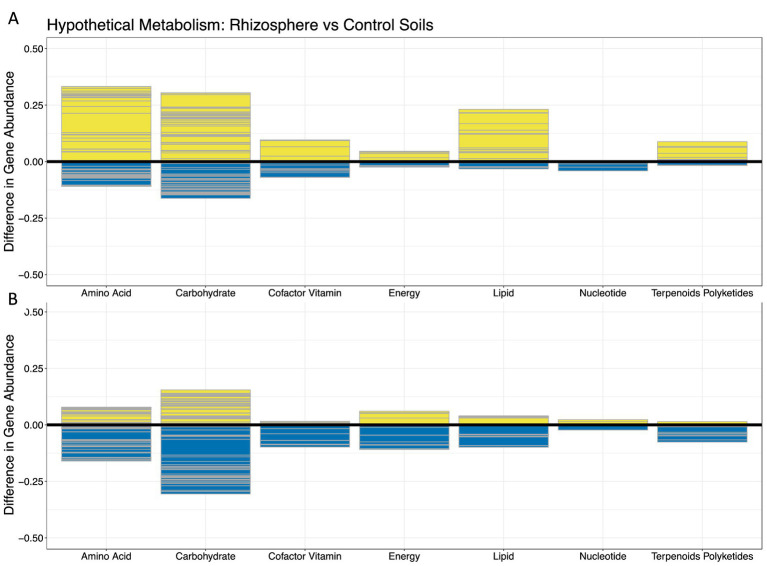
Hypothetical functional profiles of rhizosphere vs. control-bulk soil microbial communities as calculated by Tax4Fun analysis, based on **(A)** RNA-derived corncob analysis, and **(B)** 16S-ratio *t*-test analysis. Y-axis represents the difference in relative abundance of a gene within a community between enriched and depleted genera in the rhizosphere soils. X-axis represents the different categories of metabolism that identified genes were associated with. Each gray line in the barplot delineates the relative abundance of a specific gene KEGG number within the category of what that gene is able to metabolize. Differences in gene relative abundances above zero (yellow) represent genes which were more abundant in the “genera enriched in rhizosphere soils” category, and the values below zero (blue) represent genes which were more abundant in the “genera depleted in rhizosphere soils” category.

The genera of the RNA-derived community characterization that showed differential abundances between the rhizosphere and control soil environments were assigned hypothetical functional profiles using the software Tax4Fun (Aßhauer et al., 2015), as were the genera deemed by convention significantly different between soil environments using 16S-ratio analysis *T*-tests (Figure 6; Supplementary Table S2). Tax4fun predicts functional capabilities of microbial communities using read abundance of 16S datasets, and returns what percentage of a community is associated with a particular gene, which relates to a metabolic capability.

In rhizosphere vs. control soils, profiles of hypothetical metabolism between the corncob and 16S-ratio *t*-test genera do follow some similar patterns. For example, the corncob analysis indicated a higher proportion of the community of genera enriched in the rhizosphere is dedicated to amino acid and carbohydrate metabolism compared to other forms of metabolism (Figure 6A), and the 16S-ratio *t*-test agrees, though it reverses which of these categories are more prevalent (Figure 6B). Genera derived from corncob analysis (Figure 6A) also indicate that lipid metabolism is prevalent in the rhizosphere compared to control soils, which we do not see in the 16S-ratio derived soils (Figure 6B). Genes associated with the metabolism of various compounds such as galactose were highly abundant in the rhizosphere soils compared to control soils, in both the corncob and 16S-ratio *t*-test analysis. Genes associated with fatty acid degradation were only visibly prevalent in the corncob genera analysis (Figure 6).

## Discussion

The goal of this study was to determine whether protein synthesis potential (RNA) microbial community profiles provided more insight than total microbial membership (DNA) into the differences between the rhizosphere soils of *B. stricta*, control soils of similar composition to rhizosphere soils, and bulk soils taken directly from the study site. Simultaneous nucleic acid extraction of DNA and RNA was performed for each sample, and 16S rRNA-transcripts (RNA) and 16S rRNA-genes (DNA) were used to create amplicon libraries. Bacterial communities generated using DNA and RNA reads were assembled for comparison, and RNA community analysis was compared to normalized 16S-ratio (RNA/DNA) community analysis. This study revealed distinct differences between the overall RNA- and DNA-derived community composition regarding OTU identity and absolute and relative abundance, which corresponds to previous studies (Denef et al., 2016; Li et al., 2019) (Figures 3, 4). The magnitude of differences between the three different soil communities varied depending on what data (DNA which tells microbial membership; RNA which tells PSP) was used for analysis.

When comparing the overall DNA- and RNA-derived communities, differences in read number and unique taxa were observed, and these differences were consistent with those reported on in other studies (Mikkonen et al., 2014; Schostag et al., 2015; Klein et al., 2016; Gill et al., 2017; Bowsher et al., 2019; Li et al., 2019, p. 201). The higher number of RNA- to DNA-derived reads likely reflects the higher abundance of ribosomes to gene copies within a cell (Moeseneder et al., 2005), which within a single prokaryotic cell can range from 800 to 35,000 in a *Vibrio* sp. (Flärdh et al., 1992), 200–2,000 in a *Sphingomonas* sp. (Fegatella et al., 1998) and 6,700 and 72,000 in *coli* (Dennis and Bremer, 1974), while gene copy numbers usually vary from 1 to 15 copies per genome (Schaechter et al., 1958; Cooper and Helmstetter, 1968; Klappenbach, 2001; Lee et al., 2009). The taxa unique to the RNA-derived community [16 OTU and 2,695 reads (<0.1% total reads)], also known as phantom taxa, can occur due to cells with a low gene copy number left undetected in DNA-derived analysis but a high ribosome copy number able to be detected in RNA-analysis being present in the samples (Moeseneder et al., 2005), or due to methodological differences such as biases caused by reverse transcription of RNA but not DNA (Zhen et al., 2015). The taxa unique to the DNA-derived community [45 OTU with 5,668 reads (<0.1% total reads)] likely represented genes from dead or lysed cells, free extracellular DNA, or dormant cells with low ribosomal counts (Hamilton et al., 1968; Lorenz and Wackernagel, 1987; England et al., 2004; Bakken and Frostegård, 2006).

More biologically meaningful differences in diversity between the DNA- and RNA- derived microbial community profiles are also consistent with other studies, which have found significant divergence in PSP and microbial membership in soils (Baldrian, 2019). Rhizosphere soils were significantly different in Shannons and Simpsons Diversity Indices between the DNA- and RNA-derived community profiles, indicating a difference between microbial membership and PSP within samples (Figure 2). These differences were further emphasized by the significant divergence in DNA- and RNA-derived community compositions (Figure 5A), which were not proportional between phyla in many cases (Figures 3A, 4). Six Phyla were significantly more abundant in the DNA than the RNA communities, with Saccharibacteria and Gemmatimonadetes seen as significantly more abundant in DNA even after the communities had been normalized to relative abundance, which accounts for differences due to read count differences between the RNA- and DNA-derived communities. This indicates these two phyla have low PSP compared to cell presence within the soil community. Differential abundance analysis further supports these differences, showing 66 genera to be lower in abundance in the RNA- than the DNA-derived community, implying lower PSP levels in those 66 genera, among which Gemmatimonadetes were included (Figure 1A). Taxa with low PSP levels are likely not active in the microbial community, despite being prevalent in terms of total microbial membership, which can affect analyses of microbial community functions and effects on soil environment biochemical cycles (Aira et al., 2010). Phyla significantly more abundant in the RNA- than the DNA-derived community, such as proteobacteria (Figures 3, 4) have a higher level of PSP for the number of cells detected in the community, while phyla such as Bacteriodites which are not significantly different between the RNA- and DNA-derived communities have an equivalent contribution to the PSP of the community to what their microbial membership would indicate.

When examining the bacterial communities of the rhizosphere, control, and bulk soil environments, some potential environmental interactions become visible when analyzing the RNA-derived community, which are not apparent in the DNA-derived community. Differential abundance analysis showed more genera to differ significantly between soil types in the RNA- than the DNA-derived community (Figure 4), indicating that these genera have different levels of potential activity within the microbial community than what is implied by microbial membership alone. Of note, Comamonadaceae, Rhizobacter, and Variovorax, known root associates of grasses and key members in sulfur cycling (Schmalenberger et al., 2008), were enriched in the rhizosphere, but went undetected in the DNA-derived community analysis. These genera were also detected to be significantly different between soil types in 16S-ratio T-tests (see Supplementary Tables S1, S2). It is not unexpected for these genera to have been found to be highly active at the field site, as Crow Creek is surrounded by pine trees and some local grasses. However, that we are seeing elevated levels of PSP in the *B. stricta* rhizospheres implies that while the cell presence of these genera remained relatively unchanged between the rhizosphere and control soils, some factor in the rhizosphere environment caused their PSP levels to rise compared to control soils.

While it is interesting to note that cyanobacteria have a very high PSP for the number of members in the community, indicated by 16S-ratio (Figure 3B), they make up such a low number of reads in the RNA and DNA derived communities to be insignificant in differential abundance analysis between soil types (Figures 3, 4), and, on average, are insignificant in terms of 16S-ratio *t*-tests as well (Supplementary Table S2). Looking solely at the 16S-ratio community analysis, cyanobacteria appear to be major players in the soil, however by comparing the 16S-ratios to the overall RNA-derived community we can see how the importance of the phylum is overestimated when determining differences between the three soil types in terms of PSP and total microbial membership.

In order to gage how the two methods of measuring significant levels of PSP (RNA-derived community profile differential abundance analysis and 16S-ratio t-test) affected analysis of microbial community functions, Tax4Fun was utilized to generate hypothetical functional profiles of the genera deemed significantly different between rhizosphere and control soils (Figure 6). We see similar overarching patterns where genes associated with the metabolism of amino acids and carbohydrates are most prevalent in the rhizosphere soils compared to the control soils. The RNA-derived community also indicates genes associated with fatty acid degradation to be prevalent in rhizosphere soils, which is not indicated by the 16S-ratio analysis (Figure 6). Carbohydrates and lipids are a known category of root exudates, and include organic compounds such as sugars and fatty acids (Vives-Peris et al., 2019). The prevalence of these genes may reflect the rhizosphere environment driving the abundance differences of ribosomes in these genera between different soil types. The higher prevalence of these genes in the rhizosphere, as indicated by both 16S-ratio and RNA-derived community analysis, could reflect resource utilization strategies of root exudates by bacteria. Confirming whether genes associated with metabolism are present in the microbial community, and whether root exudates are being metabolized by microbes would need further exploration using more comprehensive metabolomic and metagenomic techniques. However, preliminary research such as this study can provide jumping off points for more in depth questions.

In this study we showed that using protein synthesis potential along with total microbial membership is a useful tool in understanding soil microbial communities. The RNA-derived community analysis indicated that bacteria which may be insignificant in the DNA-based community analysis may have more of an effect on the overall microbial community functional profile. In terms of determining which genera are significantly different between soil treatments, RNA-derived analysis provides more information than DNA-based analysis, as RNA reflects how microorganisms are interacting with the different soils, exposing more fine-scale differences in the communities between treatments and indicators of which genera are driving the majority of soil community function. How the RNA-derived data is analyzed, whether by using 16S-ratios or by analyzing the RNA reads as a community describing protein synthesis potential rather than microbial membership, changes which genera are defined as significant between soils as well as levels of relative activity. Using corncob differential abundance analysis may provide a more accurate reflection of the community PSP than 16S-ratios, as corncob analysis reduces analyses biases inherent in microbial data. When using PSP to create hypothetical functional profiles of the microbial communities, the genera identified by corncob to be significantly different between soils revealed more about the rhizosphere community potential functions in a biologically relevant way than 16S-ratio significant genera. Future studies into the effects of the local soil microenvironment on microbial community composition and function should take PSP into account, as fine-scale differences between the rhizosphere and surrounding bulk soils can be lost or underestimated when only total microbial membership is observed. Understanding the fine-scale influences the plant rhizosphere environment has on the soil microbial community is important for researchers looking to optimize plant-microbiome relationships to maximize plant health and fitness for agricultural purposes, as well as for environmental scientists researching the feedback systems between plants and local soil microbiomes.

## Data Availability

The original contributions present in the study are publically available, and can be found at: Ceretto, Alessandra; Weinig, Cynthia, 2025, “A Comparison of 16S rRNA-gene and 16S rRNA-transcript Derived Microbial Communities in Bulk and Rhizosphere Soils”, https://doi.org/10.15786/OPEXCU, Wyoming Data Repository, V1.

## Footnotes

^1^ https://doi.org/10.15786/M2FY47
